# Relational continuity of oral health care in Indigenous communities: a qualitative study

**DOI:** 10.1186/s12903-019-0986-z

**Published:** 2019-12-23

**Authors:** Richa Shrivastava, Yves Couturier, Stefanik Simard-Lebel, Felix Girard, Nadia Verenna Bendezu Aguirre, Jill Torrie, Elham Emami

**Affiliations:** 1 Faculty of Dentistry, Université de Montréal, Montréal, Québec H3C 3J7 Canada; 2 School of Social Work, Université de Sherbrooke, Sherbrooke, J1H 4C4 Québec Canada; 3 Director of Specialised Services, Cree Board of Health and Social Services of James Bay, Mistissini, Québec G0W 1C0 Canada; 4 Faculty of Dentistry, McGill University, Montréal, Québec H3A 1G1 Canada

**Keywords:** Continuity of patient care, Primary health care, Integrated health care systems, Oral health, Indigenous population

## Abstract

**Background:**

The relational continuity of care is an essential function of primary health care. This study reports on the perspectives of Cree communities and their primary health care providers regarding the barriers and enablers of relational continuity of oral health care integrated at a primary health care organization.

**Methods:**

A multiple case study design within a qualitative approach and developmental evaluation methodology were used to conduct this research study in Cree communities of Northern Québec. Maximum variation sampling and snowball techniques were used to recruit the participants. Data collection consisted of individual interviews and focus group discussions. Thematic analysis was conducted which included transcription, debriefing, codification, data display, and interpretation. The consolidated criteria for reporting qualitative studies (COREQ) were used to guide the reporting of study findings.

**Results:**

A total of six focus group discussions and 36 individual interviews were conducted. Five major themes emerged from the thematic analyses for barriers (two) and enablers (three). Themes for barriers included impermanence and lack of effective communication, whereas themes for enablers included culturally competent professionals, working across professional boundaries, and proactive organizational engagement.

**Conclusions:**

Based on these findings, relational continuity can be empowered by effective strategies for overcoming barriers and encouraging enablers, such as recruitment of permanent professionals, organizing cultural competency training, development of a Cree language dental glossary, encouraging inter-professional collaboration, and promoting the organization’s efforts.

## Background

For almost four decades, primary health care has been introduced as a gateway that can address the equitable, population-centred service delivery and needs of a complex health care system such as some Indigenous health care organizations [1]. This approach puts emphasis on prevention, community involvement, and a multi-sectoral approach in which the continuity of care plays a central role [1]. Moreover, promoting integrated care is essential in improving continuity of primary health care services [2, 3]. Successful models of integrated care have employed various aspects of continuity of care [4]. Continuity of care has been defined as *the degree to which a series of discrete health care events is experienced by people as coherent and interconnected over time and consistent with their health needs and preferences* [4, 5]. Three types of continuity of care have been discussed in the literature: information, management, and relational continuity [6]. Reid et al. provided the following definition of relational continuity (also known as interpersonal continuity): *an ongoing relationship between patients and providers is the undergirding that connects care over time and bridges discontinuous events* [6]. Relational continuity has been highly valued by patients and linked with higher patient satisfaction and better outcomes [7–9]. It is considered one of the important attributes of primary health care as it is associated with improved communication, trust, empathy, and interpersonal relationship [10–12].

Furthermore, the relational continuity of care as a facilitator of primary health care [13] has been put under the spotlight by the Five Foundations for Integrated Care model, elaborated by the Canadian Nurses Association, the Canadian Medical Association, and the Health Action Lobby [11]. It appears in the vision of patient’s medical home presented by the College of Family Physicians of Canada [14], as well as in some implementation studies [13, 15, 16]. According to the World Health Organization report on integrated people-centred health services, approaches to attaining relational continuity include providing consistent care by the same providers, developing continued relationship and trust among providers and patients, and adapting care to patients’ social, cultural, and psychological factors [4]. Relational continuity is also improved by the work of dedicated coordinators, such as case managers or navigators [17].

Historically, relational continuity has been inferred most commonly from the degree to which patient care is concentrated in a single physician [13]. However, new models of primary health care internationally and in Canada are moving towards inter-professional team-based care, which may strengthen relational continuity [4, 18]. Furthermore, oral health care disparities due to the geographic isolation of rural and remote communities may also be addressed within the new definition for relational continuity.^4^

Worldwide, Indigenous populations constitute a higher proportion of rural and remote populations and experience significantly poorer health and oral health [19]. These disparities are attributed to multifactorial social determinants including socio-economic, historical, cultural, dietary and lifestyle changes, geographical and health service systems [20, 21]. Approaches and interventions to deal with these disparities include culturally tailored community-based initiatives, integrated dental services into primary health services, incorporating traditional practices into dental care and training of health care professionals [20, 21]. These approaches enables relational continuity by community empowerment, better communication and availability of information, developing more trust and confidence, and minimizing discrimination [4].

For instance, geographical isolation and historical and social inequalities resulting from historical colonization and assimilation policies compromised the engagement in a patient-provider relationship especially for these Indigenous populations and interrupted cultural safety [19]. In fact, one of the essential elements in achieving relational continuity is adapting health care to the patient’s cultural beliefs [4]. In culturally safe health care services, health professionals are expected to understand the traditional and historical background of their patients and to integrate this towards their provision of care for their patients [22]. This approach can empower the relational continuity of care in any health care setting [23, 24].

Hence, it is important to assess relational continuity in the evaluation of health care services. Therefore, the objective of this study was to answer this research question: What are the perspectives of patients, primary health care providers, and administrators at an Indigenous health care organization regarding barriers and enablers of relational continuity of oral health care integrated within an Indigenous primary health care organization?

## Materials and methods

### Study design

This study is nested in a study funded by Canadian Institutes of Health Research entitled: “Oral Health Integrated into Primary Care: Participatory Evaluation of Implementation and Performance in Quebec Cree Communities” [25]. This participatory study used a qualitative approach to get a deeper insight into the perception^6^ and lived experiences commonly shared by the study participants in regard to relational continuity of care. We used a multiple case study design within a qualitative approach and developmental evaluation methodology [26, 27]. Developmental evaluation is useful in learning and adapting a complex intervention or program to the emerging conditions in collaboration with the key stakeholders [26]. The case study is an appropriate research design in evaluating and exploring a new or unclear phenomenon within its real-life setting [27].

### Study setting

Nine Cree communities of Northern Québec are geographically situated across 450,000 km^2^, a vast area including nine villages with a population of nearly 18,000. The Cree Board of Health and Social Services of James Bay (CBHSSJB) is responsible for the administration of appropriate health and social services in these communities [28, 29]. The CBHSSJB has developed two Strategic Regional Plans 2004–2014 and 2017–2021 [29] that proposed implementing a model for the integrated delivery of health and social services in the Cree communities including the integration of oral health with primary care [29, 30]. In all communities, CBHSSJB operates Community Miyupimaatisiiun (Wellness) Centres (CMC) that are responsible for general medicine, home care, dentistry, and social services through a team of primary health care providers, along with one regional hospital primarily serving the largest community [31]. Regionally, they have developed a 29-bed Regional Hospital. In these communities, the dental clinic is integrated with the CMC or regional hospital [31]. The dental care providers offer public dental services at the clinic. Moreover, the dental hygienists along with community health representatives provide preventive dental care to the age group 0–9 years via school, daycare, and CMC [31].

### Ethical considerations and informed consent

Ethical approval was obtained from the Institutional Review Boards of the Université de Montréal and McGill University, and the research committee of the Cree Board of Health and Social Services of James Bay. This research is compliant with OCAP (ownership, control, access, and possession) principles for conducting research involving First Nations communities [32]. Written informed and oral consent was obtained from the study participants.

### Data collection

We purposefully selected four communities in terms of demographic, geographical, cultural, and health and oral health care characteristics to achieve maximum heterogeneity. Using the participative approach, the community health care providers were involved in all aspects of the study including study participant recruitment using snowball technique and maximum variation sampling [33, 34]. Participants included patients, health care providers, and administrators at CBHSSJB in the four selected communities. Data collection included face-to-face individual interviews and focus group discussions (FGD) which were conducted in a private setting at the CMCs and regional hospital. These data were collected by the teams of two researchers trained in qualitative research. There was no existing relationship between the interviewers and the participants. An interview guide with open-ended semi-structured questions was developed based on the study’s conceptual framework, the Five Foundations for Integrated Care [11], which was then tailored based on the study participant’s profile (patients, administrators, health care providers). The five foundations for integrated care include patient access, patient-centered care, management continuity of care, relational continuity of care, and informational continuity of care [11]. This study was focused on an in-depth investigation of the relational continuity of care.

### Data analysis

Data analysis included transcription, debriefing, codification, data display, thematic analysis, and triangulation [35]. All the interviews and focus group discussions were audio-recorded and transcribed verbatim. We employed an inductive thematic analysis approach using ATLAS.ti 8 software and segmented data to analyze the relational continuity of integrated primary oral health care. Initial codes were generated followed by allocation of these codes to potential themes**.** Multiple potential themes were identified and grouped under overarching themes for discussion [36]. Transcription, coding, and thematic analyses were conducted by two research trainees (RS, SLL) followed by cross-examination and investigator triangulation of data by a lead researcher (EE) to enhance the quality of the findings. The thematic analysis was revised by the nominated community stakeholders and other team members. The recommendations outlined in the Consolidated Criteria for Reporting Qualitative Studies (COREQ) were utilized to guide the reporting of our findings [37].

## Results

A total of six focus group discussions and 36 individual interviews were conducted with 74 health care providers, patients, and administrators. Table 1 presents the profile of the study participants. Two major themes for barriers and three for enablers were developed from the thematic analyses. To ensure confidentiality, numbers have been used for the identification of participants.

**Table 1:** Profile of the study participants. Total 45 participants in focus group discussions and 36 in personal interviews

S.No.	Participants Categories	Number of participants^a^	Gender distribution		Ethnicity	
			Male	Female	Cree	Non-Cree
1	Dental health care workforce	20	4	16	9	11
2	Community health care providers	26	4	22	16	10
2	Administrators	18	7	11	9	9
3	Patients	10	1	9	9	1

^a^A total of seven participants were involved in both individual interviews and focus group discussions

The themes for barriers of relational continuity of care were impermanence and lack of effective communication.

### Impermanence

Relational continuity progresses gradually with time and develops trust and confidence in patients towards health care providers. Participants identified the shortage of permanent dentists and their continuous transitions as a barrier and a challenge for the relational continuity of care at the different levels of the health organization. Yet some communities have enough permanent dental health care providers; participants mentioned their shortage as a challenge to relational continuity.

*He’s [dentist] only here temporarily [. . . .] It’s difficult for [the dental team] to always readjust to everybody coming and going [. . .] and the population also feel the movement, […] so it’s difficult for them to trust the dental team overall.* (Administrator 1, FGD)

They also mentioned that the impermanence of the dental health care providers may be the source of distrust of care, on the part of patients. Development of distrust was noteworthy in the case of providing dental services to children.

*The first dentist I had, she explained to me that I need a cleaning [. . . .] I relied on that, but she’s not here anymore! And then, I’ve seen another dentist [. . .] he had to fix cavities and he didn’t proceed in my last dentist’s schedule, like I was supposed to get this cleaning.* (Patient 1, interview)

*But I know this, my children are [. . .] sometimes they afraid when there is new dentist . . . so, ya, there is a difference [in the approach]. I think [. . .] they don't know the children. (*Patient 2)

Participants desired to have permanent dentists not only to establish individual patient–provider trusted relationship but also to achieve positive health impacts at the community level.

*May I’ve got the same dentist since thirty years [. . .] . And I wouldn’t change . . . . Because he got all of my things and he follow-up on my things [. . .] . When it’s permanent staff, the population is better because she is being to be known by the nurse, by the doctor. When it’s permanent staff, the population is always better served.* (Primary health care provider 1, FGD)

Although the patients’ desire was to improve relational continuity with the presence of permanent dentists, the shortage of dental workforce in the primary organization hindered this need because of the priority for receiving care rather than investing in relational continuity of care.

*I told [. . .] you can just call me up my extension, you know just call me whenever there is an opening, I could just run there [. . .] cancel what I am doing and just run there*. (Primary health care provider 2, interview)

### Lack of effective communication

Participants linked impermanence to communication gap for the incoming health providers. This communication gap affects the smooth coordination of patient’s follow-ups. In this situation, new care providers may require additional support in navigating this transition phase and in maintaining smooth communication and coordination.

*The fact that people go and come, doesn’t make it easy. And there is not necessarily [. . .] a follow-up on the person that left, had that kind of things going on so when the other people will come and replace, … they should keep following up on that, so it’s just to keep the communication going but yeah , we have to have a link with them at all times.* (Patient 3, interview)

Lack of effective bidirectional communication between patients and health care providers was also expressed as an element of relational continuity. The communication in local language facilitates the transmission of information and understanding among community residents. The language barrier was expressed as a major barrier of the relational continuity of care especially in case of children and elderly patients.

*They don’t give us much information when you just talk to them in English. But when you communicate in Cree, it’s easier for them.* (Primary health care provider 3, interview)

*The communication is not there, and they understand very, very limited [. . .] their understanding is basically yes and no.* (Patient 4, interview)

*I think one of the things [. . .] is the communication, is the language, yes Cree is the predominant language here [. . .] and sometimes the clients come in [. . .] they have their language barrier between the dentist and whatever staff and yet maybe some of them learn in school but don’t want to speak the language [. . .] and that’s what we see, we have it in kids, we have it in the adults that don’t want to speak [. . .] that don’t have the full understanding of the English language [. . .].* (Administrator 2, interview)

Lack of all the technical dental terms in the local language was expressed as another challenge to better communication: “*nobody has ever done any work on language in dental to look at what Cree terms are, equivalent for English terms*” (Administrator 3, FGD).

**Themes for enablers of the relational continuity** included culturally competent professionals, working across professional boundaries, and proactive organizational engagement.

### Culturally competent professionals

The presence of local and Indigenous health care providers was identified as instrumental for a culturally competent communication and continuity of care. These local health care providers act as a link between non-Indigenous care providers and patients by translating the communication among them whenever required. They act as the *gatekeeper of the continuity of care* by maintaining the continuity of services in case of professional turnover.

*When there is little kids and they don’t speak English or French or when elders they don’t speak French or English and [local health care provider] are here to explain, you’re gonna translate. And I think this is really a key factor.* (Dental care provider 1, FGD)

*They [Indigenous dental secretary or dental assistant] are the bridge between the dentist and the patient, so [. . .] they are Cree and the message goes much faster and easier, because it’s in the cultural context, it’s much easier to message to transmit easier, like if I tell a patient that you need to brush more, the message sometimes doesn’t go through as quick, but if it’s by them, it’s faster.* (Dental care provider 2, interview)

### Working across the professional boundaries

Participants valued interprofessional teamwork and coordination for health care services in the organization. They mentioned that dental health care providers, primary health care providers, and community health representatives were collaboratively working on the patient’s care to improve relational continuity of care.

*It [trust] comes with time, it depends on how you’re gonna interact with the assistant, with the secretary, with the hygienist, with the patient, you know with everyone.* (Dental care provider 1, FGD)

*Well I do the teachings even if the pamphlets are not there, from what I remember, I do teach the parents to really work on the health of the child.* (Primary health care provider 3, interview)

*We have a very strong team, team works as an interdisciplinary team and they collaborate very well with each other and within the different services that they provide.* (Primary health care provider 4, interview)

### Proactive organizational engagement

The organization is taking initiatives to maintain the relational continuity of care and fill the communication gaps. The organization has the provision of appointing replacement dentists in case of absence of working dentists as a step to fill the gaps in care continuity.

*Sometimes we have “permanent replacing dentist” [. . .]. That’s like a replacing dentist only doing replacement. We are calling them permanent replacing dentists as they are coming often in the same community, but they are replacing the permanent dentists [. . .]. So [. . .] they can build, and they have built the trust between patients.* (Dental care provider 1, FGD)

Other initiatives included organizing meetings for new care providers and developing a guideline for replacements and new dentists that helps them to understand their role and work culture at the clinic.

*It is a job description, it is the same document we use as a guideline for dentist when they arrive, and they are new. And it gets a little bit better, but then with time, we lose them, and we have someone new and it’s hard to keep the same balance. But at least with that new tool [that is] provided to all the new dentists that come in the territory and it’s basically a guideline without putting any name on the forms: what goes well, what does not, what improves, what needs to be worked on more*. (Administrator 1, FGD)

*When we have new workers, we have a meeting with them [. . .] we talk with them about this is the way you fill the file, etc.* (Dental care provider 3, interview)

## Discussion

Relational continuity is co-created between the patients and primary health care providers characterized by their shared understanding, good communication, and addressing patients’ needs in a coordinated way [38]. The results of our study highlighted the importance of professional permanence, communication, inter-professional coordination, and culturally competent care providers in a proactive health care organization that positively favours relational continuity. Some of the barriers in this study, such as lack of workforce, long waiting list, and language barriers, and enablers such as cultural competency, were similar to those previously found on the issue of access to care in rural and remote communities [39–41].

As per Rainbow model of integrated care in a primary health care setup, integration extends from clinical integration (micro level), professional and organizational integration (meso level) and system integration (macro level), with normative and functional integration as enablers across all levels [2, 42]. Our results identified elements of clinical, professional, organizational,normative and functional integrations as enablers of relational continuity in this Indigenous integrated primary health care setup [2] .

Several studies have been conducted to evaluate the impact of relational continuity of care on health and health care outcomes in various health care domains including long term conditions and chronic diseases such as diabetes and asthma [43–47], special needs health care [48–50], and maternal health care [15]. However, to the best of our knowledge, this study is the first to explore the perspective of a northern Indigenous community on relational continuity in terms of oral health care in a primary care organization.

Relational continuity and professionals’ permanency:

Consistency of professionals is considered as an influencer for relational continuity in establishing a continued patient–provider relationship with a sense of trust and affiliation, patient–provider mutual understanding, and better quality of consultations [51]. Similar to previous studies, our results have also identified that turnover of health care providers and inconsistent staffing jeopardize relational continuity at the organizational level [19, 52]. Previous studies also support our results that trust and confidence with a physician develop over time [53–55]. The higher turnover of health care providers results in dissatisfaction, isolation, or helplessness and confusion due to receiving different treatment and medical advice [46, 51]. Furthermore, the insufficiency of professionals negatively affects relational continuity due to unavailability of an appointment at a suitable time and long waiting times for appointments, which hampers service utilization [56] .

Relational continuity and doctor-patient communication:

Evidence on doctor–patient communication linked effective communication with improved health care quality measures including patient satisfaction, treatment compliance, and health outcomes, which in turn can improve the relational continuity of care [57]. In the present study, we found barriers to effective communication between the oral health provider and patients especially in children and elderly patients. An ineffective communication due to language barriers has been associated with frustration and feeling of neglect and isolation among patients [56]. The presence of an interpreter not only improves the quality of the patient–provider conversation but also provides more eloquent explanations of the patient’s condition [56]. This is observed as an enabler for primary oral health care integration in the present study as local health care providers were noticed taking this opportunity. However, non-Indigenous primary health care providers in these communities need to understand historical, sociodemographic, political, and cultural contexts of rural Indigenous health and health inequities to provide effective relational continuity of care [19].

Relational continuity and interprofessional collaboration:

Our results are consistent with the WHO report suggesting that relational continuity facilitates effective care coordination in an integrated health care setting [4]. Shared responsibility and collaboration among dental care and primary care providers are instrumental in promoting the integration of oral health care [58]. This was observed to be an enabler of relational continuity, and further efforts will be required to stimulate ongoing communication by defining, implementing, and evaluating each health provider’s specific role.

Recommendations:

Based on the study results, the following strategies are recommended by the participants to overcome barriers and promote enablers in Cree First Nations and other northern multi-lingual communities:

- Health care policies and practical strategies based on financial and non-financial incentives (such as spousal employment opportunities, further education and training opportunities, social needs incentives such as housing, transport, and care and education of children) to promote the recruitment of permanent health care providers.
- Custom training of local/ Indigenous people to become professional health care providers.
- Encouraging better integration of health care workers from outside by promoting effective communication with the development of specific cultural competency training for health care professionals to learn, in this case, the Cree language, to better understand local traditions.
- Engaging, in this case, the Cree communities and elders to collaborate in the development of a Cree language dental glossary to help improve communication.
- Encouraging inter-professional health care collaboration by providing education and training in oral health care and by recruiting trained Dental Community Health Representatives to act as a link between the different health sectors.

Limitations and research implications:

In the long term, progressively working on providing relational continuity in an Indigenous health care organization could help in improving inequitable oral health service delivery in rural Indigenous communities [19]. We believe that our results and recommendations can be applied to other Indigenous health care organizations. Qualitative exploration in this study enabled us to gather in-depth information on relational continuity in integrating oral health care in an Indigenous primary health care organization from patients’, health care providers’, and administrators’ perspectives. However, due to the nature of qualitative studies, the study results may not be generalizable or directly transferable to other international health care settings. Further longitudinal studies are needed to understand factors associated with ongoing relational continuity of oral health care at a primary health care organization.

## Conclusion

At CBHSSJB, impermanence of dental health providers and lack of effective communication skills in local language were key barriers in providing relational continuity of care; however, cultural competence of health care providers and team working across primary health services appear as major enablers. Based on the study findings, relational continuity can be empowered by effective strategies for overcoming barriers and encouraging enablers such as recruitment of permanent professionals, organizing cultural competency training, encouraging inter-professional collaboration, and promoting the organization’s efforts.

## Data Availability

The data sets generated and analyzed in this study are available by contacting corresponding author on reasonable request.

## Abbreviations

CBHSSJB: Cree Board of Health and Social Services of James Bay
CMC: Community Miyupimaatisiiun (Wellness) Centres
COREQ: Consolidated criteria for reporting qualitative studies
FGD: Focus group discussions
